# Should tumor VEGF expression influence decisions on combining low-dose chemotherapy with antiangiogenic therapy? Preclinical modeling in ovarian cancer

**DOI:** 10.1186/1479-5876-6-2

**Published:** 2008-01-08

**Authors:** David O Holtz, Robert T Krafty, Alisha Mohamed-Hadley, Lin Zhang, Ioannis Alagkiozidis, Benjamin Leiby, Wensheng Guo, Phyllis A Gimotty, George Coukos

**Affiliations:** 1 Center for Research on Early Detection and Cure of Ovarian Cancer, University of Pennsylvania, Philadelphia, USA; 2 Division of Gynecologic Oncology, Department of Obstetrics and Gynecology, University of Pennsylvania, Philadelphia, USA; 3 Abramson Family Cancer Research Institute, University of Pennsylvania, Philadelphia, USA; 4 Center for Clinical Epidemiology and Biostatistics, University of Pennsylvania, Philadelphia, USA; 5 Department of Biostatistics and Epidemiology, University of Pennsylvania, Philadelphia, USA

## Abstract

Because of its low toxicity, low-dose (LD) chemotherapy is ideally suited for combination with antiangiogenic drugs. We investigated the impact of tumor vascular endothelial growth factor A (VEGF-A) expression on the efficacy of LD paclitaxel chemotherapy and its interactions with the tyrosine kinase inhibitor SU5416 in the ID8 and ID8-Vegf models of ovarian cancer. Functional linear models using weighted penalized least squares were utilized to identify interactions between Vegf, LD paclitaxel and antiangiogenic therapy. LD paclitaxel yielded additive effects with antiangiogenic therapy against tumors with low Vegf expression, while it exhibited antagonism to antiangiogenic therapy in tumors with high Vegf expression. This is the first preclinical study that models interactions of LD paclitaxel chemotherapy with antiangiogenic therapy and tumor VEGF expression and offers important lessons for the rational design of clinical trials.

## Introduction

Expansion of vasculature is critical for tumor growth. Tumors cannot grow beyond few millimeters in the absence of angiogenic support provided by vascular endothelial growth factor-A (VEGF-A or VEGF) and other soluble factors [1]. Approaches to block tumor angiogenesis have therefore attracted significant attention, and combination of antiangiogenic therapy targeting VEGF with standard cytotoxic chemotherapy has provided proof of principle in the clinic [2-5]. Low-dose (LD) or metronomic chemotherapy was designed to damage tumor endothelial cells through the close, regular administration of low, nontoxic doses of chemotherapeutic drugs with short drug-free intervals [6-8]. Additionally, LD chemotherapy suppresses angiogenic factors and inhibits the recruitment and function of circulating endothelial progenitor cells and/or circulating endothelial cells [9-11]. Based on preclinical studies and early phase clinical trials, despite lower cumulative doses, clinical efficacy of LD chemotherapy may be superior to maximally tolerated dose regimens [12-18]. Because of its low toxicity, LD chemotherapy is ideally suited for combination with other drugs, including antiangiogenic drugs. LD chemotherapy enhanced the effects of antiangiogenic therapy in preclinical models [19] and has yielded encouraging results in combination with antiangiogenic drugs in the clinic [6,20,21].

In spite of its obvious promise, translation of LD chemotherapy to the clinic faces numerous challenges, including defining a biologically optimal dose, and identifying the optimal drug for combination schemes in specific disease settings, since combination therapy with antiangiogenic drugs has proven beneficial in some patients but not in others. To date it is unclear what factors determine the likelihood of success of LD chemotherapy with antiangiogenic therapy.

VEGF is a critical angiogenic factor in advanced ovarian carcinoma [22]. Expression of VEGF varies considerably among tumors of similar origin, and VEGF overexpression at the mRNA level or increased serum levels portend poor survival [22-24]. Upregulation of VEGF by tumor cells may be in response to metabolic starvation or hypoxia [25], but may also be constitutive as a result of oncogene amplification [26]. To date, the effect of VEGF expression on tumor response to LD chemotherapy has not been investigated. Furthermore, it remains unknown whether tumor VEGF overexpression influences tumor response to combination of antiangiogenic therapy and LD chemotherapy. Yet, these are important questions that may affect clinical decisions.

We investigated the impact of tumor Vegf on the efficacy of LD chemotherapy, and examined whether tumor Vegf affects the interactions of metronomic chemotherapy with an antiangiogenic drug. As examples of LD chemotherapy we used paclitaxel, a drug commonly used in ovarian and other cancers, while as an example of antiangiogenic therapy we used SU5416, a tyrosine kinase inhibitor with activity against VEGF receptor-2 (VEGFR-2) [27,28], which has been used in the clinic in combinations [6]. We used the ID8 and ID8-Vegf mouse models of ovarian cancer to address the above questions [29]. ID8 cells express constitutively low levels of Vegf-A, while ID8-Vegf cells were retrovirally transduced to express constitutively high levels of Vegf_164 _isoform. This model recapitulates closely human ovarian cancer. We have shown that ID8-Vegf tumors maintain significantly higher levels of Vegf-A expression *in vivo*; exhibit increased angiogenesis and growth; and are associated with significantly shorter survival relative to ID8 tumors. Importantly, tumor, ascites and serum levels of Vegf-A protein in animals bearing ID8-VEGF tumors were significantly higher (approximately 28, 13 and 3-fold, respectively) than in animals bearing control ID8 tumors, but both were within the range described in human ovarian cancer [29]. To analyze the interactions between tumor Vegf, LD chemotherapy and SU5416, we used a novel method of statistical modeling that involves fitting functional linear models using weighted penalized least squares [30,31]. This approach enabled us to investigate interactions between LD chemotherapy and antiangiogenic therapy through simple experiments.

We found a significant difference in tumor response depending on Vegf expression. LD chemotherapy yielded additive effects with antiangiogenic therapy only against tumors with low Vegf expression, while it exhibited antagonism to antiangiogenic therapy in tumors with high Vegf expression. This is the first preclinical study that models interactions of LD chemotherapy with antiangiogenic therapy and tumor Vegf expression and offers important lessons for the rational design of clinical trials.

## Materials and methods

### Cell culture and reagents

The development and characterization of ID8-Vegf cell line was described elsewhere in detail [29]. ID8 and ID8-Vegf cells were maintained in DMEM media (Invitrogen, Carlsbad, CA) supplemented with 10% fetal bovine serum (FBS), 100 U/ml penicillin, and 100 μg/ml streptomycin (Roche, Indianapolis, IN) in a 5% CO_2 _atmosphere at 37°C.

### Mice and treatments

Six to eight week old female C57BL/6 mice (Charles River Laboratories, Wilmington, MA) were used in protocols approved by the IACUC of the University of Pennsylvania. Mice were treated with intraperitoneal (i.p.) bolus injections of pacitaxel, SU5416 or dimethyl sulfoxide (DMSO). Paclitaxel (7.5 mg/kg in 0.2 ml 0.9% saline) or saline alone were given i.p. on days 1 and 4 every week. This dose is approximately one-fourth of maximally tolerated doses (MTD) for mice [32,33] and are within metronomic range. SU5416 (20 mg/kg in 25 μl DMSO, Sigma-Aldrich, St. Louis, MO) or DMSO (25 μl) alone were given i.p. on days 1, 3, and 5 every week. This dose of SU5416 and DMSO are MTD for C57BL/6 mice, as were identified by dose-defining experiments in healthy 6 week old female C57BL/6 mice (not shown). Higher doses of SU5416 or DMSO resulted in significant weight loss or mortality.

### Tumor-free Matrigel™ experiments

In some experiments, mice (n = 3 mice/group) were anesthetized with 20 μl/gm tribromoethanol/tert-amyl alcohol solution (Avertin) and injected with 0.5 ml Matrigel™ (BD Biosciences, Bedford, MA) containing recombinant mouse (rm)Vegf_164 _(100 ng/ml). One day after Matrigel™ injection, mice were treated with i.p. paclitaxel and/or SU5416 or DMSO at the above doses. Paclitaxel or saline were given i.p. on days 1 and 4. SU5416 or DMSO were given on days 1, 3, and 5. Matrigel™ plugs were removed under anesthesia 7 days later and were snap frozen in liquid nitrogen.

### Tumors

Subconfluent ID8 or ID8-VEGF cultures were trypsinized, washed twice, and cells harvested by centrifugation at 1,000 *g *for 5 min. A single-cell suspension was prepared in PBS mixed with an equal volume of cold Matrigel at 10 mg/ml. A total volume of 0.5 ml containing 1 × 10^6 ^ID8 or ID8-Vegf cells was injected subcutaneously (s.c.) into the flank of 6-week old C57BL6 mice (*n *= 10). Tumors were detectable two weeks later and tumor size was measured weekly thereafter using a Vernier caliper. Tumor volumes were calculated by the formula *V *= 1/2 (*L *× *W*)^2^, where *L *is length (longest dimension) and *W *is width (shortest dimension) [34]. Treatments were initiated fourteen days after tumor inoculation and were carried out for 6 weeks. After listed time periods, mice were euthanized, and tumors were removed and snap frozen in liquid nitrogen.

### Tumor microvascular density

Snap frozen tumors and Matrigel plugs were stored at -80°C; embedded in OCT compound (Sakura Finetek, Torrance, CA); frozen in liquid nitrogen; and cut with a cryostat into 8 μm sections. For immunofluorescent staining, sections were sequentially incubated in 5% horse serum; biotin-labeled anti-mouse CD31 antibody (1:400, BD Bioscience); and avidin-FITC or avidin-Cy5 (BD Bioscience). All sections were imaged using an upright Nikon (Augusta, GA) E-600 Eclipse microscope equipped with a Bio-Rad (Hercules, CA) 1024-ES confocal system. Images were acquired through Cool SNAP Pro^® ^color digital camera (Media Cybernetics). All tumors were viewed at ×200 magnification. For Matrigel plugs, CD31 staining was analyzed using Image-Pro^® ^Plus 4.1 software (Media Cybernetics). For microvessel density measurements, slides were scanned at low power (x40) to identify areas of highest vascularity. Ten high-powered (x200) fields were then selected randomly within these areas and microvessel densities were calculated based on the number of CD31-positive structures. At least three tumors from each group were examined in three sections. Sections from each tumor were separated by at least 200 μm.

### Statistical methods

A fixed effects analysis of variance was used for between group comparisons with the *in vitro *data and with the ECM data observed after 6 weeks of treatment. Means are reported with their standard errors. To assess interactions between LD paclitaxel and antiangiogenic therapy without assuming any specific functional form for tumor volumes over time (such as assuming that the natural logarithm of tumor volume over time is linear), we fit a functional linear model via weighted penalized least squares that included covariates for treatment with paclitaxel, treatment with SU5416, ID8-Vegf, and all subsequent two- and three-way interactions [30,31]. By not a priori assuming a form for the shape characterizing the changes in tumor volume over time, this smoothing spline based approach differs from a standard linear regression by avoiding the bias induced by deviations of the data from a nice parametric form and by allowing for the assessment of interactions over time Smoothing parameters used in the algorithm to estimate the parameters in the functional linear model were selected using generalized maximum likelihood, while 95% Bayesian confidence intervals and likelihood ratio tests were used to perform inference [35-37].

## Results

### Low-dose paclitaxel inhibits neovasculature formation in tumor-free Matrigel

**Figure 1:**
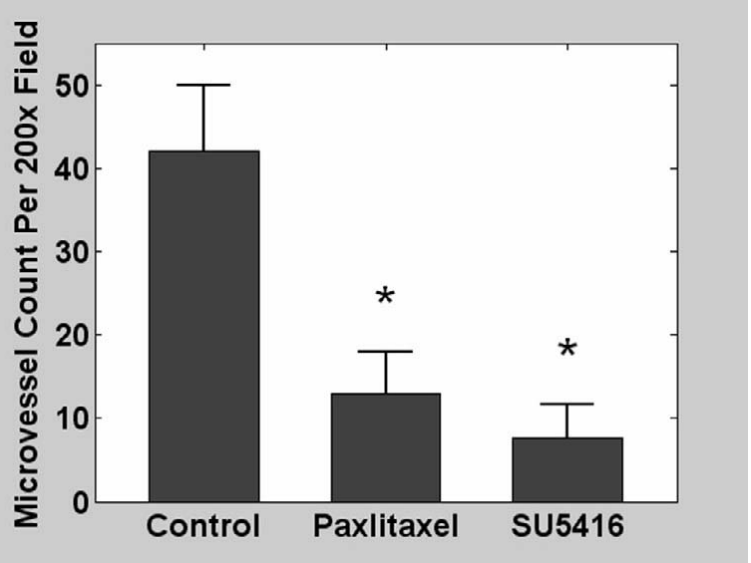
Average microvessel count in tumor-free Matrigel plugs in mice treated with phosphate buffered saline and DMSO (control); low dose paclitaxel; or SU5416. Matrigel plugs were enriched with recombinant mouse Vegf_164 _(100 ng/ml). * indicates p ≤ 0.05.

The tumor-free Matrigel model has been used as a suitable tool to measure the efficacy of antiangiogenic therapy *in vivo*. We tested whether paclitaxel suppress new blood vessel formation at one-fourth of MTD, a dose similar to those used in the clinic. Healthy mice were inoculated on day 0 with tumor-free Matrigel containing rmVegf_164_. Mice were treated on days 1 and 4 with i.p. paclitaxel at 1/4 MTD. Control mice were inoculated with tumor-free Matrigel and treated with SU5416 at MTD or PBS plus DMSO on days 1, 3, and 5. Matrigel plugs were analyzed on day 7 for microvascular density. The average microvascular density at 7 days was 42 microvessels per high power field (200×) in control mice treated with PBS/DMSO (Figure 1). LD paclitaxel as well as SU5416 at MTD resulted in significant suppression of vascular development (both, p < 0.0001). Thus paclitaxel at 1/4 MTD twice per week suppressed vessel formation *in vivo*.

### Therapeutic efficacy of low-dose paclitaxel on ID8 and ID8-Vegf tumors

**Figure 2:**
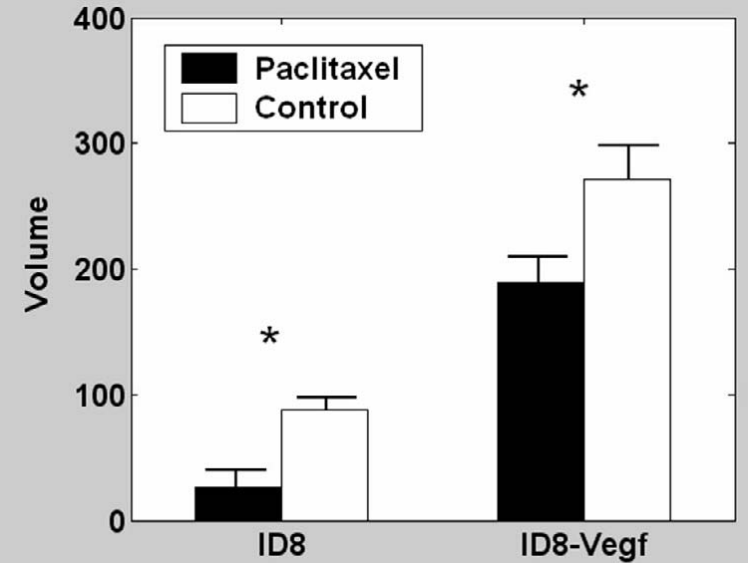
Effect of low dose paclitaxel on ID8 or ID8-Vegf tumors. Mice were treated for 6 weeks, starting 2 weeks after tumor inoculation. Tumor volumes were recorded at completion of therapy. * indicates p ≤ 0.05.

Next, we tested the effects of LD paclitaxel on tumor growth in the ID8 and ID8-Vegf model (Figure 2). Low-dose paclitaxel at the above dose and schedule had a significant inhibitory effect on the growth of ID8 tumors (Figure 2, left). The volume of ID8 tumors treated with paclitaxel (26.6 ± 13.2) was significantly smaller than control ID8 tumors treated with PBS (88.1 ± 9.6 p = 0.0029). Low-dose paclitaxel had also significant inhibitory effect on the growth of ID8-Vegf tumors (Figure 2, right). The volume of ID8-Vegf tumors treated with paclitaxel was significantly smaller (189.2 ± 20.6 than control ID8-Vegf tumors treated with PBS (271.2 ± 27.2 p = 0.026). These results show that both ID8 and ID8-Vegf tumors respond to paclitaxel.

### Interactions of SU5416 with LD paclitaxel in ID8 tumors

**Figure 3:**
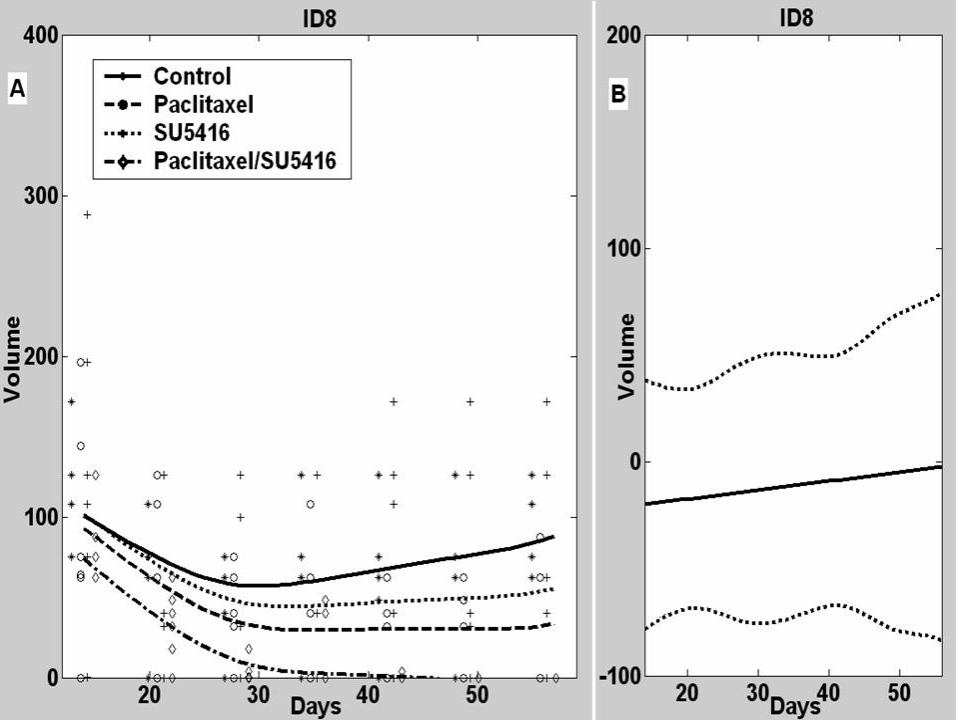
**A**, Mean tumor volumes and estimated ID8 tumor growth curves in control mice or mice treated with paclitaxel, SU5416 or their combination. **B**, Estimated difference between the observed mean tumor volume in animals receiving combined paclitaxel/SU5416 therapy and a predicted tumor volume assuming additive effects for the two individual drugs with 95% confidence intervals.

To evaluate the interaction between low-dose paclitaxel and SU5416 on tumor growth, we treated animals bearing ID8 tumors with LD paclitaxel (plus DMSO), SU5416 (plus saline), or paclitaxel plus SU5416 (Figure 3A). Control mice were treated with saline plus DMSO. As previously noted, LD paclitaxel delayed the growth of ID8 tumors (p = 0.003), while SU5416 had a nonsignificant inhibitory effect on ID8 tumors (p = 0.62). The combination of paclitaxel and SU5416 resulted in significant suppression of tumor growth where ID8 tumors treated with LD paclitaxel plus SU5416 were 13-fold smaller than control tumors (p < 0.001) and tumors became undetectable in many animals. Thus, LD paclitaxel was more efficacious than SU5416 against tumors with low Vegf expression. Furthermore, combination of SU5416 and paclitaxel was quite efficacious against ovarian carcinoma that expressed low levels of Vegf resulting in substantial tumor regression.

We used the fitted functional linear models to further characterize the interactions between SU5416 and LD paclitaxel in ID8 tumors (Figure 3B). The estimate of the difference between actual effect of combination therapy and the estimated theoretical additive effect of the two drugs on ID8 tumor volumes was approximately zero for all time points (p = 0.81), indicating that SU5416 when combined with paclitaxel had an additive effect on ID8 tumors.

### Effects of LD paclitaxel and SU5416 on microvascular density in ID8 tumors

**Figure 4:**
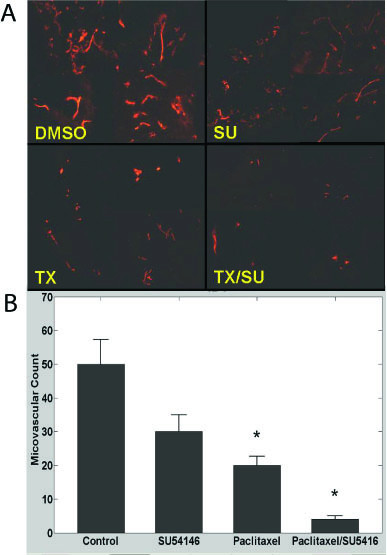
**A**, Examples of microvascular density in ID8 tumors of control mice or mice treated with SU5416, paclitaxel or combination. Vessels were visualized with CD31 immunostaining. Four tumor microphotographs are combined for each treatment. 200× magnification. **B**, Microvascular density quantification in ID8 tumors of control mice or mice treated with SU5416, paclitaxel or combination. * indicates p ≤ 0.05.

To better understand the interaction of paclitaxel and SU5416, we sought to define the effects of each drug on tumor microvasculature *in vivo*. Microvascular density (MVD) was assessed by CD31 immunostaining (Figure 4). SU5416 alone resulted in mild but not significant decrease in MVD in ID8 tumors (p = 0.4 both). Paclitaxel alone resulted in significant decrease in MVD (p = 0.05). Importantly, paclitaxel plus SU5416 resulted in marked (>90%) reduction in MVD (p = 0.03), indicating a potent drug interaction at the level of the vasculature. These results are in agreement with the effects of the drugs on tumor growth described above. Thus, LD paclitaxel alone suppressed significantly MVD and growth of tumors with low Vegf expression, and its combination with SU5416 produced more dramatic results on MVD and tumor growth.

### Interactions of SU5416 with LD paclitaxel in ID8-Vegf tumors

**Figure 5:**
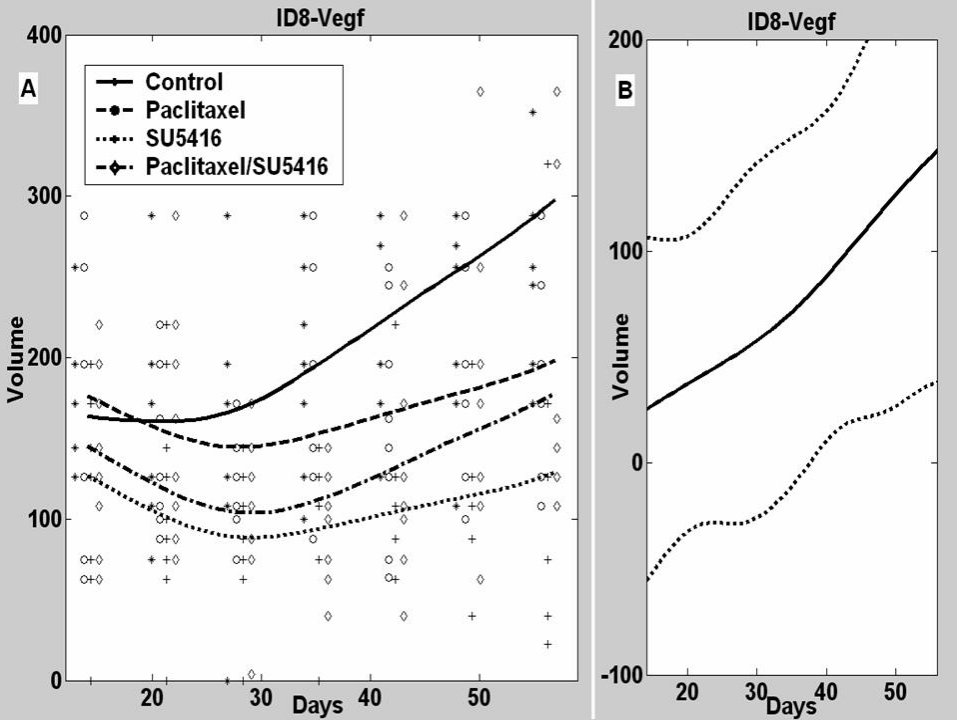
**A**, Mean tumor volumes and estimated ID8-Vegf tumor growth curves in control mice or mice treated with paclitaxel, SU5416 or combination. **B**, Estimated difference between the observed mean tumor volume in animals receiving combined paclitaxel/SU5416 therapy and a predicted tumor volume assuming additive effects to the two individual drugs with 95% confidence intervals.

Paclitaxel alone had a significant inhibitory effect on the growth of ID8-Vegf tumors (p = 0.026) (Figure 5A). SU5416 alone was similarly efficacious in inhibiting the growth of ID8-Vegf tumors (p < 0.001). The combination of paclitaxel and SU5416 resulted in significant suppression of ID8 tumor growth (p < 0.001), but was slightly less effective than SU5416 alone. Thus, the benefit of drug combination was not seen in tumors with high expression of Vegf.

We used fitted functional linear models to further investigate the interactions between SU5416 and LD paclitaxel in ID8-Vegf tumors (Figure 5B). In contrast to ID8 tumors, the difference between the observed effect of combination therapy on tumor volume and the estimate of the theoretical additive effect was positive for ID8-Vegf tumors after 37 days (p = 0.03). Thus, the combination of SU5416 with paclitaxel was less than additive (antagonistic resulting in increased tumor volume) in ID8-Vegf tumors.

### Effects of LD paclitaxel and SU5416 on microvascular density in ID8-Vegf tumors

**Figure 6:**
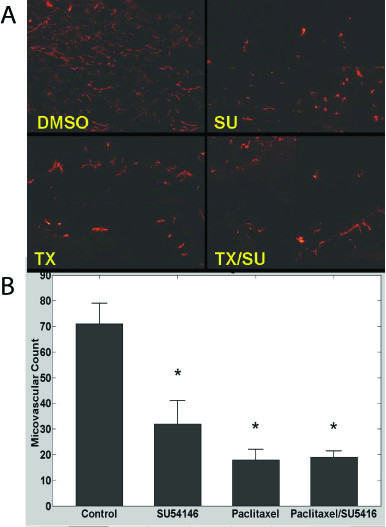
**A**, Examples of microvascular density in ID8-Vegf tumors of control mice or mice treated with SU5416, paclitaxel or combination. Vessels were visualized with CD31 immunostaining. Four tumor microphotographs are combined for each treatment. 200× magnification. **B**, Microvascular density quantification in ID8-Vegf tumors of control mice or mice treated with SU5416, paclitaxel or combination. * indicates p ≤ 0.05.

We have previously shown that Vegf overexpression results in increased tumor MVD [29]. SU5416 reduced MVD (p = 0.05) (Figure 6); LD paclitaxel alone induced a significant and more pronounced reduction in MVD (p = 0.008). Addition of SU5416 to paclitaxel did not further suppress MVD compared to paclitaxel alone. These results are in agreement with the effects of the drugs on tumor growth described above.

## Discussion

To date, the influence of tumor Vegf expression on the response to LD chemotherapy, anti-Vegf therapy or their combination has not been investigated. Our study addresses these interactions for the first time. We found that SU5416 administered alone reduced MVD and tumor growth primarily in tumors with high Vegf expression, while it was not as efficacious against tumors with low Vegf expression. In keeping with previous evidence [38], LD paclitaxel administered alone exhibited marked antitumor efficacy. Interestingly, LD paclitaxel was efficacious also against tumors with low Vegf expression, and in these tumors outperformed SU5416 used at MTD. Further investigation is warranted to understand the mechanisms underlying these differences. It is possible that tumors with low Vegf expression develop alternate, Vegf-independent mechanisms that support tumor growth and thus are resistant to Vegf inhibition. For example, tumors with mature vessels respond less to Vegf inhibition [39]. Of relevance, we have previously reported that ID8-Vegf tumors exhibit immature vessels, while ID8 tumors exhibit more mature vessels surrounded by pericytes [40]. LD paclitaxel may circumvent this limitation as it may exert toxicity on tumor vascular cells independently of maturity.

We used a statistical approach that enabled us to first evaluate the effect of combinations of therapy on tumor volume over time with simple experiments and then assess the difference between the observed effect of combination therapy and what would be expected if the two drugs had an additive effect. To the best of our knowledge, this is the first application of semiparametric functional linear models in preclinical tumor studies. This approach better approximates growth curves that do not satisfy assumptions inherent in the standard models. The examples provided in this work illustrate the power inherent in this approach in estimating the ability of two therapies to act additively or not. The additive effect of two therapies occurs when the difference between the observed effect on the tumor when both therapies are given and the predicted effect on the tumor, based on the theoretical additive effect of the two therapies given alone, approximates zero. In the case of tumor volume, a positive difference is evidence for a less-than-additive (negative) interaction or antagonism, while a negative difference indicates a greater-than-additive (positive) interaction or synergism. This analytical model has the potential to proving extremely useful in preclinical screening of drug combinations and dose optimization of metronomic chemotherapy when characteristics of the tumor microenvironment are important factors in therapeutic outcome.

Our findings indicate that combination of antiangiogenic drugs with LD paclitaxel provides therapeutic advantage in tumors expressing low levels of Vegf, while in tumors with high Vegf expression the combination did not provide any benefit or was rather antagonistic. Lack of additive effect between two drugs in tumors with high Vegf expression may signify that both drugs act through the same pathways, thus their concomitant use cannot produce more effects than either one alone. Why is however this lack of cooperation seen only in tumors with high Vegf expression and not in tumors with low Vegf expression? It is possible that only in tumors with high Vegf expression the action of each drug can be maximized. It is interesting that antiangiogenic therapy alone performed similar to LD paclitaxel in tumors with high Vegf expression. Thus, clinically either therapeutic approach could be chosen based on the desired toxicity profile.

The present results suggest that clinical investigators testing combinations of LD chemotherapy and antiangiogenic therapy should make an attempt to measure pretreatment tumor Vegf expression. However, the best way to measure VEGF in the clinic is unclear. Tissue levels of VEGF-A protein would be ideal, but this requires invasive procedures. Plasma VEGF might be a reasonable surrogate. Results from xenograft studies have indicated that plasma VEGF levels following therapy can predict outcome [41,42], while in the clinic pretreatment or post-treatment levels of serum or plasma VEGF have been correlated with outcome [22-24,42]. However, no study has yet examined plasma and tumor levels of VEGF in the human, and in a retrospective subset analysis, pretreatment tumor mRNA levels or plasma protein levels of VEGF did not correlate with benefit from the addition of bevacizumab to standard chemotherapy in pancreatic or colorectal cancer [43,44]. Further testing is required to validate our findings in the human.

The present study has specific strengths and limitations. For example, we employed a syngeneic mouse model, which allows capturing the effects of complex tumor-host interactions in an immunocompetent host. Thus, it can closely recapitulate events that may take place in ovarian cancer in the human. Future investigation in this model could help us understand whether any of the interactions observed might also involve immune mechanisms, as Vegf blockade is known to restore antitumor immune response while paclitaxel may activate immune mechanisms through Toll receptor 4 [45-47]. On the other hand, in this study we used a flank model rather than an orthotopic tumor model. This was mainly because we sought to evaluate drug interactions on microvascular density, which can be reliably interpreted only in this model, and we sought to report observations that are possibly applicable to many other solid tumor models. Future studies will address the effect of drugs on the intraperitoneal model.

## Conclusion

Our work provides the first evidence that tumor Vegf expression influences the interactions between LD chemotherapy and antiangiogenic therapy. Our results suggest that tumor Vegf expression should be measured in clinical trials testing the above approaches. LD paclitaxel is best combined with antiangiogenic therapy targeting Vegf against tumors with low Vegf expression, where such combination could achieve dramatic responses without major toxicity. Tumors with high Vegf expression, on the other hand, may likely benefit from antiangiogenic therapy as much as from LD chemotherapy, and alternate combinations need to be evaluated, including high dose chemotherapy.

## Competing interests

The author(s) declare that they have no competing interests.

## Authors' contributions

DOH carried out the in vitro and in vivo studies and drafted the manuscript and figures. RTK developed the statistical algorithms and performed the statistical analyses and drafted the statistical sections of the manuscript and some of the figures. AMH assisted with the in vivo experiments. LZ generated the mouse model used in these studies and optimized norphology methodologies. IA assisted with the in vivo experiments and data analysis. BL participated in the statistical analyses. WG oversaw development of the statistical algorithms. PAG developed statistical approach and oversaw the statistical analyses. GC conceived of the study, participated in its design and coordination, and finalized the manuscript.
